# Zooplankton communities and *Bythotrephes longimanus* in lakes of the montane region of the northern Alps

**DOI:** 10.1080/20442041.2017.1294317

**Published:** 2017-05-02

**Authors:** Zsófia Horváth, Csaba F. Vad, Christian Preiler, Julia Birtel, Blake Matthews, Radka Ptáčníková, Robert Ptáčník

**Affiliations:** ^a^ WasserCluster Lunz, Lunz am See, Austria; ^b^ Department of Aquatic Ecology, EAWAG, Kastanienbaum, Switzerland

**Keywords:** Alps, *Bythotrephes*, elevation, montane, oligotrophic, zooplankton

## Abstract

Lakes in the Alps represent a considerable fraction of nutrient-poor lakes in Central Europe, with unique biodiversity and ecosystem properties. Although some individual lakes are well studied, less knowledge is available on large-scale patterns essential to general understanding of their functioning. Here, we aimed to describe crustacean zooplankton communities (Cladocera, Copepoda) and identify their environmental drivers in the pelagic zone of 54 oligotrophic lakes in the montane region of the Alps (400–1200 m) in Austria, Germany, and Switzerland, covering a spatial scale of 650 km. Moreover, we aimed to provide data on the distribution and ecological requirements of the North American invader *Bythotrephes longimanus* in its Central European native range. Communities were mainly dominated by widespread species typical of lowland habitats, and only a few true specialists of oligotrophic alpine lakes were present. The most frequent taxa were the *Daphnia longispina* complex and *Eudiaptomus gracilis*, with 48 and 45 occurrences, respectively. Species richness decreased with altitude and increased with lake area. The main structuring factors of community composition were chlorophyll *a* concentration and depth, which drove an apparent separation of mesotrophic and oligotrophic communities. *Bythotrephes* had 13 occurrences, showing a preference for deep oligotrophic lakes. Its presence was not coupled with lower crustacean species richness, as was repeatedly observed in North America. Additionally, it frequently co-occurred with the other large predatory cladoceran, *Leptodora kindtii*. *B. longimanus* might be considered a truly montane species in Central Europe, given its absence in lowland and alpine lakes.

## Introduction

In oligotrophic lakes, nutrients are the primary limiting factor for biomass production. Such nutrient-poor lakes can be numerous in the boreal and mountainous regions. In these lakes, plankton communities are relatively simple and species-poor, yet zooplankton–phytoplankton interactions are stronger than in eutrophic lakes (McQueen et al. 1986), and plankton in general plays a central role in ecosystem functioning (Straškrabová et al. 1999, Callieri et al. 2002, Sarnelle and Knapp 2005).

Compared to their boreal counterparts (Rühland et al. 2003, Lepistö et al. 2004, Hessen et al. 2006, Walseng et al. 2006, Ptacnik et al. 2008, 2010), little is known about regional patterns of plankton diversity in alpine lakes (Anderson 1971, Knapp et al. 2001, Reche et al. 2005, Tolotti et al. 2006), mostly due to the remoteness and low accessibility of lakes in mountainous areas (Straškrabová et al. 1999, Sommaruga 2001). Studies of alpine lakes more often emphasise localised patterns of diversity and environmental change, for example in paleolimnological studies (Wolfe et al. 2001, Nevalainen et al. 2014) or long-term monitoring programs of a few chosen lakes (e.g., Ruggiu et al. 1998, Straile 2000, Anneville et al. 2004, Pomati et al. 2015). A regional-scale perspective on the diversity of alpine lakes is greatly needed, especially for monitoring and predicting the large-scale effects of environmental changes, particularly climate change, to which diversity and functioning of these ecosystems are highly sensitive (Anneville et al. 2004, Holzapfel and Vinebrooke 2005, Blenckner et al. 2007, Manca et al. 2007, Parker et al. 2008). Aquatic habitats in mountainous areas are especially perceptive to changes in both precipitation and temperature regimes (Sommaruga-Wögrath et al. 1997, Sommaruga et al. 1999), which can induce significant changes in the biomass and composition of plankton communities of these lakes (e.g., Sommaruga et al. 1999, Holzapfel and Vinebrooke 2005, Shatwell et al. 2008).

Within the plankton communities, crustacean zooplankton is a key component of lake ecosystems, linking energy flow from bacterioplankton and phytoplankton to higher trophic levels, such as fish. Zooplankton communities are strongly influenced by environmental conditions within a lake (such as temperature, trophic state and fish stocks; e.g., Hessen et al. 1995), with the additional influence of dispersal effects and lake history (e.g., Shurin 2001, Forrest and Arnott 2007).

Predatory cladocerans occupy a special position among crustacean zooplankton because they are both driven by environmental factors similar to those that drive other zooplankters (e.g., temperature, acidity, fish predation; Brooks and Dodson 1965, Garton et al. 1990, Sarvala and Halsinaho 1990, Herzig 1995, Vogt et al. 2013) and yet can play a significant structuring role to communities of their zooplankton prey (Herzig and Auer 1990, Yan et al. 2002, Barbiero and Tuchman 2004). Among them, *Bythotrephes* gained considerable attention after invading lakes in North America, first spreading rapidly throughout the Laurentian Great Lakes in the late 1970s and 1980s (Sprules et al. 1990), and soon afterward to surrounding inland lakes (Yan et al. 1992), and are currently established in ~150 lakes in Canada and the United States (Kerfoot et al. 2016). This invasion has, in some instances, caused lasting changes in the zooplankton communities of the affected lakes (e.g., Yan et al. 1992, Barbiero and Tuchman 2004). The invasion has initiated numerous studies on the ecology of this species in North America, whereas we know far less about the species in its native range.

*Bythotrephes* is native to northern Eurasia and has a disjunctive distribution, occurring in northern Europe (British Isles, Denmark, Scandinavia, northern Germany, Poland, Baltic countries, Belarus, and Russia) and in the Alps in Central Europe (Flössner 1972, Ketelaars and Gille 1994). Such an arctic–alpine distribution is typical for ice age relicts; the Alps today provide a cold interglacial refugium for these species in Central Europe (Stewart et al. 2010). Although recent morphological studies suggest that *Bythotrephes* consists of at least 5 closely related and relatively young species (Korovchinsky 2015, Litvinchuk and Litvinchuk 2016), molecular research showed that genetically they are all a single polymorphic species, *B. longimanus* (Therriault et al. 2002, Colautti et al. 2005). All of these studies agree, nevertheless, that populations in the alpine region certainly belong to *B. longimanus* s. str.

Other than a review from Gaviria-Melo et al. (2005), mostly based on old data from Austria, little is known about the current distribution of *B. longimanus* within the Alps. Some data are available from lakes with long-term monitoring programs (e.g., Lake Constance, Mondsee, Laggo Maggiore, and some Swiss lakes; Dokulil et al. 1990, Enz et al. 2001, Palmer et al. 2001, Manca et al. 2007), but it is unclear how widespread the species actually is in this area or whether any changes have occurred in response to ongoing environmental changes. Although *Bythotrephes* in North America seems to be successful at spreading to new localities, within Europe, a new occurrence and permanent establishment outside its native range has to date been documented only in artificial lakes in the Netherlands and Belgium in the late 1980s (Ketelaars and Breemen 1993).

Furthermore, for the alpine region, no systematic studies have been conducted on the environmental preferences and co-occurrence of this species with other zooplankton across a range of lakes, unlike for other areas of *Bythotrephes* occurrence, such as Norway (Hessen et al. 2011), the Commonwealth of Independent States (Grigorovich et al. 1998), Canadian Shield Lakes (Boudreau and Yan 2003, Weisz and Yan 2010), or selected European and North American lakes (MacIsaac et al. 2000).

Our aims were to describe the crustacean zooplankton communities of lakes in the montane zone of the northern fringe of the Alps and identify the main environmental drivers of species richness and community composition. In addition, we aimed to update the view on the current distribution of *Bythotrephes* in relation to the local environment and investigate its possible effect on the zooplankton communities in these lakes with its native occurrence.

## Methods

### Sample collection and analysis

**Figure 1.**
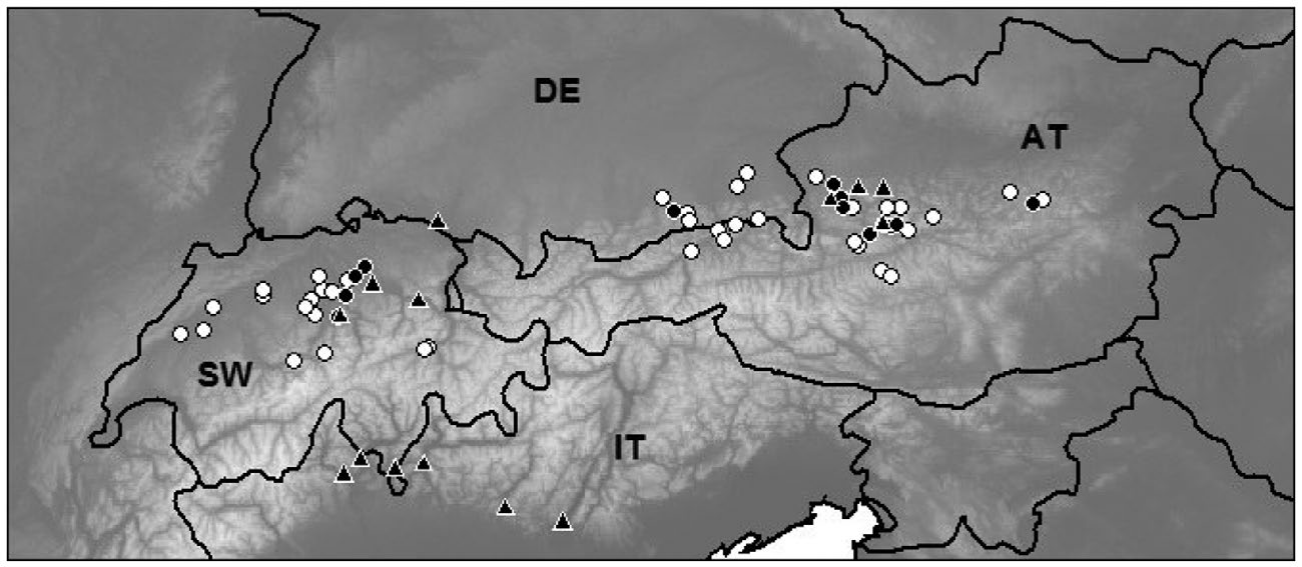
Distribution of lakes in our 2011–2012 sampling campaign (circles, *n* = 54). ● = presence of *Bythotrephes longimanus*; ▲ = occurrence in other lakes, based on published data from the last 30 years (data sources in Supplemental Table S6). Country identification: SW = Switzerland, DE = Germany, AT = Austria, IT = Italy. Background greyscale shows elevation, with white as high and darker grey as low elevations.

We sampled 54 lakes in Switzerland (*n* = 20; EAWAG sampling, summer and autumn 2011), Germany, and Austria (WasserCluster sampling, summer and autumn 2012; *n* = 8 and 26, respectively), with most of the lakes situated in the montane region of the Alps (400–1200 m; Table 1, Fig. 1).

**Table 1.** Environmental parameters of the 54 lakes in the Alps.

	Abbrev.	N	Min	Max	Mean	SD
Altitude (m)	Alt	50	406	1891	695.4	300.2
Chlorophyll *a* (μg L^−1^)	Chl-*a*	50	0.8	37.5	5.9	7.3
Mean temperature in the upper 3 m (°C)	T	41	9.1	23.1	17.0	3.3
Conductivity (μS cm^−1^)	Cond	49	16.7	443.4	236.4	85.6
Total phosphorus concentration (μg L^−1^)	TP	31	1	16.2	5.3	3.4
Total nitrogen concentration (μg L^−1^)	TN	31	204	2741	678.8	406.1
Secchi disk transparency (m)	*Z*_S_	50	0.6	16.3	5.4	3.5
Soluble reactive phosphorus concentration (μg L^−1^)	PO_4_	50	0.5	6	2.3	1.2
Lake area (ha)	lake_area	48	1	50	25.6	14.5
Lake volume (1000 m^3^)	lake_vol	43	0.01	667 070	58 296	149 969
Max depth (m)	*Z*_max_	47	2	261	54.9	55.1

Sampling was carried out at the deepest spot of small, shallow lakes (based on a bathymetric map or Google satellite picture) and at a location with a depth of >15 m in large, deep lakes.

During sampling of German and Austrian lakes, a depth-integrated water sample was taken with a 1 m tube sampler covering the whole epilimnion (surface to metalimnion, based on the temperature profile). The mixed sample was used to measure concentrations of chlorophyll *a* (Chl-*a*), total phosphorus (TP), soluble reactive phosphorus (PO_4_), and total nitrogen (TN). For Chl-*a*, we used fluorometry with aceton extraction (Arar and Collins 1997), without correcting for phaeophytin. TP was measured by using persulfate digestion (Clesceri et al. 1999) followed by the ascorbic acid colorimetric method (Hansen and Koroleff 1999). For PO_4_, the same ascorbic acid method was used for water samples filtered on muffled and acid-washed GF/F filters. TN was digested according to Clesceri et al. (1999), and afterward the automated hydrazine reduction method was used with the continuous flow analyser (Clesceri et al. 1999). In the Swiss lakes, Chl-*a* was extracted with ethanol and measured with spectrophotometry, whereas the ascorbic acid method was used for PO_4_ (Hansen and Koroleff. 1999).

Zooplankton was sampled in the German and Austrian lakes with a 100 μm mesh size (diameter: 40 cm) plankton net, for most lakes by taking a vertical net haul of the upper 15 m water layer. In shallow lakes, the net was towed from a few meters above the sediment, and in deep lakes (epilimnion thickness >15 m), the net was hauled from 25 m depth. In cases of low zooplankton abundance, an additional net haul was performed. All samples were fixed with ethanol (70%). In the Swiss lakes, zooplankton were analysed from samples collected for molecular analyses (not part of the present study) with a 100 μm mesh sized net (diameter: 50 cm) hauled through the entire water column (maximum 140 m in Lake Zurich); zooplankton were then concentrated and stored frozen until analysis, when they were transferred to 70% ethanol.

All crustacean zooplankters (Cladocera, Copepoda) were identified to species (apart from the *Daphnia longispina* complex, which was treated as one species, *D. longispina*) based on the keys of Flössner (1972, 2000), Einsle (1993), Gulyás and Forró (1999), Hołyńska and Dahms (2004), and Petrusek et al. (2005). In the Austrian and German samples, density was enumerated by subsampling 10% of the total concentrated sample, after which the whole sample was checked for rare species. In the Swiss zooplankton samples, only presence–absence was recorded by screening the entire sample because of the differences in collection methods.

### Statistical analyses

For the statistical analyses, altitude (Alt), conductivity (Cond), Secchi disk transparency (*Z*_S_), lake volume (lake_vol), and concentrations of Chl-*a*, TP, and TN were ln transformed, whereas maximum depth (*Z*_max_) was square root transformed to minimise residuals. We focused on lakes situated in the calcareous northern fringe of the Alps. There were 2 non-alkaline lakes with low Cond (<50 μS cm^−1^; Riesachsee and Schwarzensee Sölk), 1 lake with high TP (>15 μg L^−1^, Spitzingsee), and 1 lake situated at high altitude (>1200 m; Melchsee; Supplemental Table S1); these 4 lakes were excluded from all analyses involving environmental predictors.

Among trophic predictors (TP, TN, PO_4_, Chl-*a*), TP and TN data were not available from the Swiss lakes; therefore, for the analysis of the predictors of species number (carried out for the whole dataset, *n* = 50), we included only PO_4_ and Chl-*a* concentrations, which were not correlated (*r* = −0.06, *p* = 0.70). We also excluded *Z*_S_ from the analyses because of its high correlation with Chl-*a* (*r* = −0.72, *p* < 0.001; Supplemental Table S2). We also reduced the correlated lake size parameters (lake area, lake volume, *Z*_max_) to 2 uncorrelated predictors, lake area and *Z*_max_, *a priori* (Supplemental Table S2 and Fig. S1) to avoid possible overfitting.

We used multiple linear regression models to test which environmental parameters affect species richness (*n* = 50). We built 3 separate models for total zooplankton, cladoceran, and copepod species numbers. We performed stepwise model selection by Akaike information criterion (AIC) with 1000 permutation steps, using “both” as the mode of stepwise search.

To study the effect of environmental predictors on the zooplankton community, we applied a canonical correspondence analysis (CCA). Here we analysed Austrian and German lakes only (without the outliers previously listed, 3 of which were Austrian or German, resulting in *n* = 31) because more environmental predictors together with density data were measured in this dataset than in the Swiss dataset (Supplemental Table S3). Because of the large variation in zooplankton densities, data were square root transformed prior to analysis. In the null model, we included Alt, lake area, *Z*_max_, TP, TN, *Z*_S_, Chl-*a*, and Cond as environmental predictors. Significant predictors were selected with the ordistep function (direction: both; number of permutations: 1000) of the R package vegan (Oksanen et al. 2012). We carried out the analysis both with and without singletons (11 of the 30 species), but significant predictors were the same in both cases. We present here the ordination without singletons; including them would make the centroid of the ordination plot less readable, and, moreover, the species-specific results are more reliable for species with multiple occurrences.

Co-occurrence of species with >5 occurrences was analysed with a probabilistic model by Veech (2013; cooccur function of cooccur package in R) using the presence–absence matrix of 54 lakes. The algorithm calculates the observed and expected frequencies of co-occurrence between each pair of species and classifies all possible species pairs as positively, negatively, or randomly associated.

We studied the effect of local environment on the abundance of *Bythotrephes* in Austrian and German lakes (*n* = 31) based on stepwise model selection by AIC of multiple linear regressions (1000 permutation steps, with ‘both’ as the mode of stepwise search). Apart from the environmental predictors previously used for the CCA, we also included here the total density of other zooplankters (square root transformed) to represent the potential prey abundance. We made a model prediction based on the 2 most important environmental predictors. We compared models with all possible combinations of linear and generalised additive model (GAM) fitting of these 2 significant predictors based on AIC and found linear fitting to be best for both predictors. Hence, we used this model in a graphical representation of the abundance distribution of the species along the 2 most important environmental gradients, based on a surface fitting. Here the surface is represented by a contour map, whereas the empirical abundance data can be illustrated with coloured symbols.

The test was repeated on the presence–absence data of *Bythotrephes* for the entire dataset (*n* = 50) to confirm the results on the entire spatial scale of our study. We used a general linear model (GLM) with a logistic function and a stepwise model selection by AIC (1000 permutation steps, with ‘both’ as the mode of stepwise search). *Z*_max_, Alt, lake area, *Z*_S_, Chl-*a*, and Cond were included in the null model.

We ran a variation partitioning model to compare the role of local environmental factors and the possible effect of *Bythotrephes* predation on zooplankton communities. We first carried out a redundancy analysis (RDA) on the Austrian and German lakes (*n* = 31, including all species except *Bythotrephes*), in which significant predictors were selected with the ordistep function (direction: both, number of permutations: 1000). We chose RDA over CCA because the subsequent variation partitioning is based on RDA. We carried out a variation partitioning analysis on the zooplankton community, with the significant local predictors grouped together and *Bythotrephes* density as the other explanatory table.

Additionally, we tested whether the presence of *Bythotrephes* affected species richness in the lakes. We included all lakes except the 4 excluded lakes discussed earlier (*n* = 50) and ran a Welch’s 2-sample *t*-test (unequal variances were found in the *a priori F*-test), with the number of zooplankton species (excluding *Bythotrephes*) as the explained variable and the presence or absence of *Bythotrephes* as the grouping factor.

Finally, we reviewed the current occurrence of *Bythotrephes* in the Alps, including data reported in the last 30 years. All analyses were carried out with packages MASS (Venables and Ripley 2002), vegan (Oksanen et al. 2012), coocur (Veech 2013), and mgcv (Wood 2011) in R (R Development Core Team 2012).

## Results

We found 34 microcrustacean species in the 54 alpine lakes, of which 17 were copepods (4 Calanoida and 13 Cyclopoida) and 17 belonged to cladocerans. *D. longispina* (found in 48 lakes) and *Eudiaptomus gracilis* (45 lakes) were the most frequent species. Seven other species occurred in >10 lakes (*Cyclops abyssorum*, *Bosmina longispina*, *Mesocyclops leuckarti*, *Diaphanosoma brachyurum*, *Leptodora kindtii*, *Bythotrephes longimanus*, and *Ceriodaphnia quadrangula*), whereas the other 25 species were less frequent (Supplemental Table S4). *Bythotrephes* was present in all 3 countries in 13 lakes (Fig. 1).

Lakes hosted 1–12 species (mean 6.2, SD 2.1). According to the multiple linear regression models, larger lakes at lower altitudes held more species. Altitude was a significant (negative) predictor of total zooplankton, copepod, and cladoceran richness. Additionally, lake area had a positive effect on both total zooplankton and cladoceran richness. For cladoceran richness, Chl-*a* also proved to have a significant negative effect (Table 2).

**Table 2.** Significant predictors of zooplankton species richness based on stepwise model selection of multiple linear regressions.

Model	Predictor	Estimate	Std. error	*t* value	*p* value
Total zooplankton	Alt	−2.97	0.96	−3.10	0.003
	lake_area	0.03	0.02	1.84	0.07
Cladocera	Alt	−1.38	0.60	−2.28	0.03
	lake_area	0.03	0.01	2.69	0.01
	Chl-*a*	−0.45	0.18	−2.47	0.02
Copepoda	Alt	−2.06	0.62	−3.32	0.002

**Figure 2.**
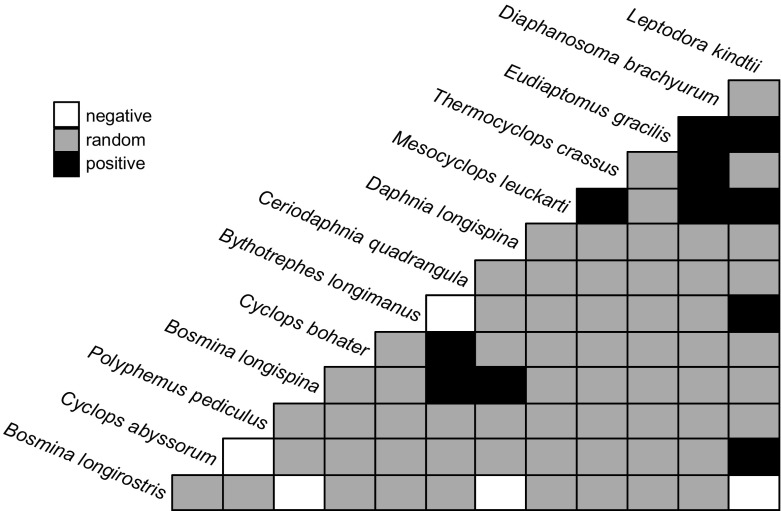
Co-occurrence matrix of species with >5 occurrences in the 54 lakes. Relationships presented as positive or negative were significant at *p* < 0.05, and random species-pair associations (*p* > 0.05) are in grey.

The 2 large predatory cladocerans, *L. kindtii* and *B. longimanus*, showed a positive co-occurrence (Fig. 2). The congeneric *Bosmina* species (*B. longirostris* and *B. longispina*) clearly separated in the lakes, with *B. longirostris* negatively and *B. longispina* positively associated with *D. longispina*. The 2 mesotrophic cyclopoids, *Thermocyclops crassus* and *M. leuckarti*, also showed a positive co-occurrence.

**Figure 3.**
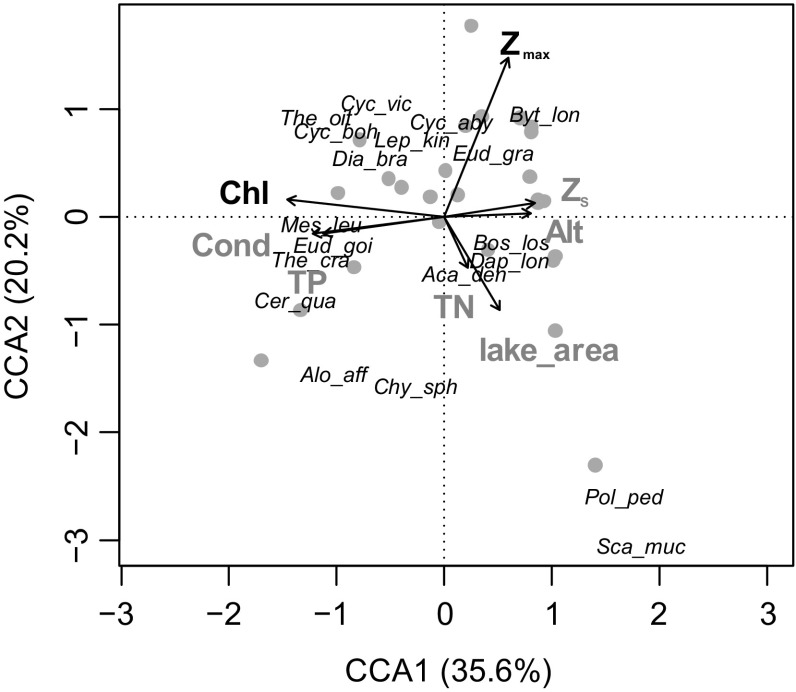
CCA ordination plot of Austrian and German lakes (significant environmental predictors in black; abbreviations of predictors in Table 1). Species abbreviations are based on the first 3 letters of genus and species names (see Supplemental Table S4), apart from *Eudiaptomus gracilis* and *E. graciloides*, which are differentiated as *Eud_gra* and *Eud_goi*).

In the Austrian and German lakes dataset, we found total zooplankton densities between 1.4 and 45.4 ind L^−1^, with most (90%) of the data <20 ind L^−1^. Chl-*a* and *Z*_max_ were identified as significant environmental predictors of the zooplankton communities (Fig. 3). The first CCA axis was strongly related to the trophic state of lakes, whereas the second axis explained variation in size (depth and area).

**Figure 4.**
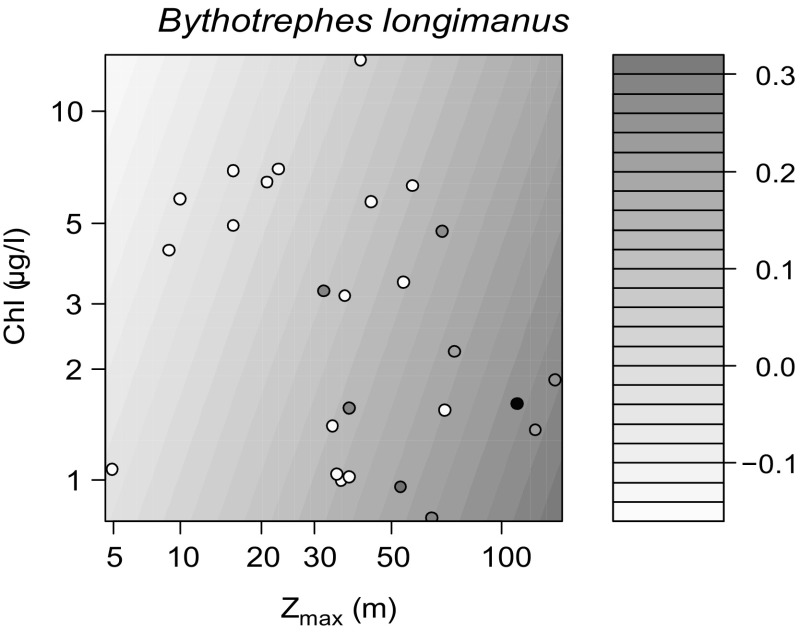
Empirical linear regression model (*R* ^2^ = 0.37) predicting the abundance (ind L^−1^) of *Bythotrephes longimanus* in the Austrian and German lakes from lake depth (*Z*_max_; *p* = 0.007) and chlorophyll *a* concentrations (Chl-*a*; *p* = 0.22), which were the strongest predictors of its occurrence (see Table 3). Points show the original empirical data, and surface is the fitted model, with darker grey as higher abundance.

In our separate analysis carried out for *Bythotrephes*, *Z*_max_ and Chl-*a* were the strongest predictors of its abundance in the Austrian and German lakes (Table 3, Fig. 4). According to the model, deep lakes with low Chl-*a* hosted this species in the highest abundances. For presence–absence data in the entire dataset (*n* = 50; results not shown), the repeated test with logistic GLM confirmed the positive effect of *Z*_max_ because it was the only significant predictor for the presence of *Bythotrephes* (*p* = 0.005).

**Table 3.** Predictors of the abundance of *Bythotrephes longimanus* in Austrian and German lakes based on stepwise model selection of multiple linear regressions (multiple *R* ^2^: 0.43).

	Estimate	Std. error	*t* value	*p* value
(Intercept)	−0.15	0.06	−2.30	0.03
*Z*_max_	0.07	0.02	3.30	0.003
Chl-*a*	−0.04	0.02	−1.89	0.07
TP	0.04	0.03	1.41	0.17

In the RDA carried out for the communities without *Bythotrephes*, the same environmental predictors proved significant, as in the CCA model built for the whole communities (Chl-*a* and *Z*_max_; ordination plot not presented). According to the results of the variation partitioning, *Bythotrephes* had no individual effect on the communities (0%, *p* = 0.6), and only the local environmental predictors had a significant individual effect (16%, *p* = 0.001), with a shared effect of 3%. Similarly, *Bythotrephes* had no significant effect on zooplankton species richness (Welch’s 2-sample *t*-test, *t* = −0.84, df = 41.42, *p* = 0.40).

In addition to the lakes visited during our survey, we found 14 instances in the literature where *Bythotrephes* has been reported in the Alps within the last 30 years (Fig. 1; Supplemental Table S6), mostly in the vicinity of Austrian or Swiss lakes involved in our analyses, with an additional group of lakes in the Italian Alps.

## Discussion

### Zooplankton communities

The lakes varied widely in TP and environmental conditions. Trophic state, in general, had a strong effect on the zooplankton communities and was a primary structuring factor for the communities and a significant predictor of cladoceran species richness. Lake trophic state is overall an important shaping factor for zooplankton (Tallberg et al. 1999, Tolotti et al. 2006, Jeppesen et al. 2011, Jensen et al. 2012). In lakes at higher altitudes than lakes in our study (in the alpine and subalpine regions of the Alps, 1800–2800 m), Tolotti et al. (2006) found the primary role of catchment characteristics, lake depth, and trophic state in shaping zooplankton communities. Trophic state indicated by Chl-*a* and TP decreased with increasing elevation in our dataset, although these relationships were not significant, neither in the whole dataset (Supplemental Table S2) nor in the subset of Austrian and German lakes (Supplemental Table S5).

The drivers of species richness in our dataset were similar to those in boreal lakes. In our study, altitude was the most important driver, with fewer species at higher altitudes. In boreal lakes, species richness showed a similar negative relationship with altitude (Hessen et al. 2006), although its effect on species richness is weaker than it was in our alpine lake dataset, with a stronger primary role of trophic state (Hessen et al. 2006). Furthermore, lake area was also found to be more important for alpine than for boreal lake zooplankton richness. We did not have data on the fish communities of the lakes, which could have also contributed to community variation, as it does in boreal lakes (Hessen et al. 2006, 2011).

The most apparent separation in our community analysis was between oligotrophic and mesotrophic species. Lakes with higher Chl-*a* and TP were characterised by *Thermocyclops oithonoides*, *T. crassus*, *M. leuckarti*, *Cyclops bohater*, and *C. vicinus* among cyclopoids, species widespread in lowland mesotrophic and eutrophic lakes (except for *C. bohater*), expanding also to lower-montane regions (Maier 1996, Nilssen and Wærvågen 2000, Jersabek et al. 2001). This finding was partly also apparent in the co-occurrence matrix, with *T. crassus* and *M. leuckarti* occurring together. Less productive lakes at higher altitude usually hosted only *C. abyssorum* among cyclopoids, which is widespread in high altitude lakes of Europe (Jersabek et al. 2001, Tolotti et al. 2006, Kernan et al. 2009).

*E. gracilis* was the most frequent copepod in the lakes, regardless of their trophic state (occurring in 45 of the 54 lakes), and has a broad geographic distribution (Riccardi and Rossetti 2007) reported from oligotrophic (Straile and Geller 1998) to hypertrophic conditions (Ponyi and Zánkai 1982). Another calanoid copepod, *Acanthodiaptomus denticornis*, was present in shallower lakes than *E. gracilis*. In Central Europe, this species is typical in both permanent and temporary habitats in the montane and alpine regions (Einsle 1993), where it occurs most frequently in upper-montane and subalpine waters between 1500 and 2000 m (Jersabek et al. 2001). This preference is the most likely explanation for the low number of encounters (*n* = 3) in our dataset.

Lower altitude, more productive lakes hosted several cladoceran species, whereas at higher elevation, only *B. longspina* and *D. longispina* were typical. Among the 3 congeneric *Bosmina* species we found, *B. coregoni* was the rarest (*n* = 3), followed by *B. longirostris* (*n* = 8), and *B. longispina* was the most frequent (*n* = 33). The latter 2 species are frequently used in paleolimnology to track changes in trophic conditions because *B. longispina* is a typical species of oligotrophic waters and *B. longirostris* is a eutrophic species (Frey 1976, Gannon and Stemberger 1978, Boucherle and Züllig 1983), which can explain the rareness of the latter species in these oligotrophic lakes.

Regional plankton biodiversity in the Alps is, in general, much less studied compared to northern European boreal lakes. Within the Alps, most of the available regional-scale knowledge on the diversity and its environmental constraints is from high-altitude alpine lakes above the treeline (Jersabek et al. 2001, Tolotti 2001, Tolotti et al. 2003, 2006), and the montane region receives even less attention. In our large-scale study covering a 650 km scale, we found that the main drivers of community composition and species richness in oligotrophic lakes in the montane zone were similar to those reported for lakes in the boreal zone (Hessen et al. 2006). The lakes were characterised mainly by widespread microcrustacean species known also from mesotrophic or eutrophic lowland habitats (e.g., *E. gracilis*, *C. vicinus*, *Thermocyclops* spp., *D. longispina*, and *L. kindtii*), together with a few alpine species (e.g., *A. denticornis*) or specialists of oligotrophic lakes (e.g., *C. abyssorum*, *B. longispina*). Therefore, regarding zooplankton communities, the montane region of the Alps represents a transitional zone between lowland lakes and the alpine lakes at higher elevations. *B. longimanus* seems to represent the only exception and might be considered a truly montane species; it is rarely found in lakes >1200 m and is not present in lowland lakes around the Alps.

### Bythotrephes longimanus *in the Alps*

Interestingly, the 2 frequent predatory cladocerans in the lakes, *B. longimanus* and *L. kindtii* showed a positive co-occurrence pattern not found previously. In Scandinavian lakes, these 2 species show a negative co-occurrence (Hessen et al. 2011), an observation based on lakes covering a much wider climatic gradient compared to those in our study, which might invoke differences. In our dataset, we found a positive co-occurrence covered habitats solely within the montane zone. Moreover, in Austria, both species prefer elevations <1600 m (Gaviria-Melo et al. 2005), suggesting that the co-occurrence pattern would be similar even if we considered higher elevation zones in the Alps.

Although it might seem unexpected that oligotrophic lakes can sustain populations of 2 large-bodied predators simultaneously, there is evidence for the effective spatial niche separation within lakes where these species co-occur, especially in oligotrophic lakes (Enz et al. 2001). In its native range, *Bythotrephes* was found to inhabit greater depths than *Leptodora* when co-occurring in the same lake (Enz et al. 2001, Palmer et al. 2001)*.* In one of the invaded Great Lakes, Lake Michigan, a similar pattern was found (Cavaletto et al. 2010). Because our samples were long vertical net hauls (sampling a water column of 15–140 m), we cannot assess their vertical separation in the investigated lakes.

Gaviria-Melo et al. (2005) reported that the highest altitude ever recorded for *Bythotrephes* is 1555 m. In our dataset, *Bythotrephes* was present between 406 and 835 m, but few lakes in our dataset were situated >1000 m. According to the observation of Gaviria-Melo et al. (2005) and Therriault et al. (2002), the species prefers deep lakes in the alpine region, a finding also supported by our analysis. It has also been reported previously that *Bythotrephes* might be sensitive to anthropogenic eutrophication, which was repeatedly implied by case studies of individual lakes (reviewed in Therriault et al. 2002). Our data show that altitude effects on *Bythotrephes* were overall weak compared to the effect of productivity, even though our data spanned an altitudinal gradient of 1000 m.

In addition to trophic state, we found that lake depth has the highest importance for the species in its native habitats. A possible explanation for this preference (apart from the previously mentioned spatial separation from *L. kindtii*) can be avoiding fish predation. Although the spiny *Bythotrephes* is protected against predation by juvenile fish (Barnhisel 1991, Barnhisel and Kerfoot 2004), it is positively selected by larger fish (Fitzmaurice 1979, Coulas et al. 1998). *Bythotrephes* was shown to exhibit diel vertical migration to avoid predation (Straile and Hälbich 2000, Young and Yan 2008), and in Lago Maggiore, its presence is enhanced by the increasing duration and thickness of the predation refuge with long-term warming (Manca and DeMott 2009).

There was only one lake (Brienzersee) in which we did not find *Bythotrephes*, although its occurrence was reported in the last 30 years (Müller et al. 2007). Nine of the 13 lakes with *Bythotrephes* in 2011–2012 represented new data on its occurrence, and we confirmed its presence in 4 lakes in which it was reported previously (Hallstättersee, Mondsee, Toplitzsee in Austria: Gaviria-Melo et al. 2005; Zürichsee in Switzerland: Enz et al. 2001).

*Bythotrephes* has been shown to have strong multiple effects on the zooplankton communities in invaded North American lakes. Previous works found that the appearance of *Bythotrephes* decreased species richness (Yan et al. 2002, Barbiero and Tuchman 2004, Kelly et al. 2013) and biomass of zooplankton (Kerfoot et al. 2016), reduced the biomass of the native predator *Leptodora* (Lehman and Cáceres 1993), altered the daytime vertical distributions of native *Daphnia* (Lehman and Cáceres 1993), and even its kairomones have nonlethal adverse effects on zooplankton (Bourdeau et al. 2016). Contrary to these effects seen in invaded lakes, we found no pronounced effects of *Bythotrephes* on the zooplankton communities in our survey. In northern Europe, Walseng et al. (2015) reported a positive effect of *Bythotrephes* on crustacean zooplankton; however, their study represents a special case because it is embedded in a spatial diversity gradient of phytoplankton and zooplankton (Hessen et al. 2006, Ptacnik et al. 2010). Likewise, Kelly et al. (2013) found that the community-shaping effect of *Bythotrephes* was much stronger in invaded Canadian lakes than in Norwegian lakes. This information collectively suggests a possibly general difference among communities co-evolved with *Bythotrephes* and communities of lakes only recently invaded by the species, which should receive more focus in future research.

## Funding

This work was supported by Austrian Research Foundation [FWF, P 26119]; Office of the Regional Government of Lower Austria; SNF [31003A-125006].

## Supplemental data

Supplemental data for this article can be accessed here. https://doi.org/10.1080/20442041.2017.1294317

## Supplementary Material

Supplementary Material
