# Supplementary material for: Zooplankton communities and Bythotrephes longimanus in lakes of the montane region of the northern Alps
Source: Inland Waters. 2017 May 2;7(1):3–13. doi: 10.1080/20442041.2017.1294317 (PMC5546047; doi:10.1080/20442041.2017.1294317)
Supplement: Supplementary Material [file tinw_a_1294317_sm4801.docx]

## Supplementary Material

## Table S1. Environmental parameters and geographic location of the 54 lakes sampled (abbreviations and units listed in Table 1 and Supplemental Table S3).

|  | Longitude | Latitude | Altitude | Chl-*a* | T | Cond | TP | TN | *Z*_S_ | PO_4_ | lake_area | lake_vol | *Z*_max_ |
| --- | --- | --- | --- | --- | --- | --- | --- | --- | --- | --- | --- | --- | --- |
| **Austria** |  |  |  |  |  |  |  |  |  |  |  |  |  |
| Almsee | 13.9575 | 47.75167 | 487 | 1.07 | NA | 145.5 | 4.14 | 664 | 2 | 1.8 | 85 | 2100 | 5 |
| Ausseer See | 13.78639 | 47.64139 | 712 | 0.96 | 11 | 127 | 2.59 | 484.5 | 9.2 | 1.53 | 210 | 72 000 | 53 |
| Erlaufsee | 15.27194 | 47.78583 | 835 | 1.57 | 15.35 | 236 | 5.69 | 847.5 | 12.5 | 1.9 | 58 | 12 296 | 38 |
| Gleinkersee | 14.29361 | 47.68778 | 804 | 5.67 | 16.1 | 199 | 7.24 | 828.1 | 4.2 | 1.8 | 13 | 10 500 | 44 |
| Grundlsee | 13.86833 | 47.63222 | 708 | 0.79 | 10 | 177 | 1.66 | 488.7 | 5.8 | 1.8 | 422 | 170 000 | 64 |
| Hallstättersee | 13.65944 | 47.57528 | 508 | 1.87 | 11.95 | 168 | 4.76 | 582.9 | 4.4 | 1.85 | 1350 | 557 000 | 125 |
| Hechtsee | 12.16333 | 47.60889 | 542 | 6.27 | 17.6 | 264 | 6 | 565.6 | 2.5 | 3.42 | 28 | 8803 | 57 |
| Hinterer Gosausee | 13.54944 | 47.5025 | 1154 | 1 | NA | 105 | 1.04 | 560.5 | 7.2 | 1.31 | 31 | NA | 36 |
| Hintersteinersee | 12.21667 | 47.53333 | 882 | 1.04 | 15.5 | 270.5 | 1.97 | 710.4 | 13.2 | 1.85 | 55 | NA | 35 |
| Hubertussee | 15.36639 | 47.80806 | 820 | 1.69 | 12.8 | 261.5 | 4.76 | 946.9 | 5.1 | 2.49 | 15 | NA | NA |
| Irrsee | 11.61806 | 47.81889 | 705 | 6.89 | 19.8 | 162 | 8.48 | 521.6 | 2.1 | 3.7 | 42 | NA | 16 |
| Krotensee | 13.38889 | 47.75278 | 538 | 2.9 | NA | 272 | 3.83 | 802.4 | 5.9 | 3.4 | NA | NA | 46 |
| Lunzer Obersee | 15.05361 | 47.85444 | 1113 | 1.24 | 11.8 | 174 | 3.83 | 620.5 | 5.5 | 1.8 | NA | NA | NA |
| Lunzer Untersee | 15.05361 | 47.85444 | 608 | 1.4 | 15.07 | 227 | 3.21 | 1145.9 | 16.3 | 1.8 | 68 | 13 600 | 34 |
| Mondsee | 13.37472 | 47.82333 | 481 | 4.72 | 15.8 | 273 | 6.62 | 487.3 | 3 | 2.06 | 1378 | 510 000 | 68 |
| Offensee | 13.83861 | 47.75361 | 649 | 1.02 | NA | 219 | 1.35 | 607.4 | 12.9 | 2.28 | 57 | 10 500 | 38 |
| Reintalersee | 11.89556 | 47.46028 | 564 | 5.78 | 19 | 329 | 8.48 | 844 | 4.1 | 4.54 | 29 | NA | 10 |
| *Riesachsee | 13.77861 | 47.32944 | 1338 | 1.49 | 9.1 | 22.2 | 3.83 | 336.5 | 9.8 | 2.65 | 18 | NA | 19 |
| *Schwarzensee Sölk | 13.87333 | 47.29306 | 1150 | 2.85 | NA | 16.7 | 3.21 | 523.4 | 5.4 | 2.65 | 23 | 2000 | 15 |
| Schwarzensee Strobl | 13.495 | 47.75222 | 716 | 3.44 | 15.1 | 224 | 8.48 | 516.9 | 5.7 | 1.85 | 48 | 13 000 | 54 |
| Steierersee | 14.03194 | 47.59917 | 1447 | 10.31 | NA | 149.5 | 4.45 | 204 | 6.7 | 2.28 | 11 | 21 | NA |
| Toplitzsee | 13.9278 | 47.6417 | 718 | 1.61 | NA | 145 | 2.59 | 583 | 7.2 | 1.42 | 54 | 33 700 | 103 |
| Vorderer Gosausee | 13.50778 | 47.52806 | 933 | 1.55 | 12.3 | 127.5 | 2.28 | 578.2 | 16.3 | 1.31 | 52 | 24 700 | 69 |
| Walchsee | 12.325 | 47.64583 | 655 | 6.42 | 16.4 | 263 | 12.21 | 788.8 | 6 | 3.53 | 95 | 11 783 | 21 |
| Wallersee | 13.1225 | 47.96667 | 503 | 6.96 | 16.7 | 285 | 8.48 | 685.6 | 3.2 | 3.34 | 6 | 76 100 | 23 |
| Wolfgangsee | 13.38889 | 47.75278 | 538 | 1.37 | NA | 211 | 1.66 | 562 | 6 | 3.37 | 14 | 667 070 | 113 |
|  |  |  |  |  |  |  |  |  |  |  |  |  |  |
| **Germany** |  |  |  |  |  |  |  |  |  |  |  |  |  |
| Brunnsee | 12.4425 | 47.98611 | 530 | 4.08 | 18.7 | NA | 5.07 | 2741 | 7.2 | 3.53 | 6 | 502 | 20 |
| Kirchsee | 11.61806 | 47.81889 | 705 | 6.89 | 19.8 | 162 | 8.48 | 521.6 | 2.1 | 3.7 | 42 | NA | 16 |
| Klostersee | 12.4425 | 47.98611 | 530 | 4.89 | 19.1 | 223 | 11.28 | 469.9 | 4.1 | 1.85 | 47 | 2762 | 16 |
| Langbürgner See | 12.35528 | 47.89972 | 530 | 3.16 | 18.9 | 244 | 4.76 | 602.6 | 5.8 | 1.9 | 104 | 9091 | 37 |
| Schliersee | 11.86222 | 47.72472 | 776 | 13.74 | 17 | 234 | 8.48 | 365.5 | 1.7 | 3.64 | 222 | 53 130 | 41 |
| *Spitzingsee | 11.88667 | 47.66611 | 1084 | 18.2 | 13.9 | 216 | 16.24 | 630.7 | 2.5 | 4.27 | 34 | 2049 | 16 |
| Tegernsee | 11.7325 | 47.72833 | 726 | 2.23 | NA | 287 | 3.83 | 665.4 | 4.2 | 3.5 | 890 | 323 090 | 73 |
| Weitsee | 12.5611 | 47.6828 | 753 | 4.2 | NA | 257 | 4.14 | 622.8 | 7.6 | 3.41 | 6306 | 2217 | 9 |
|  |  |  |  |  |  |  |  |  |  |  |  |  |  |
| **Switzerland** |  |  |  |  |  |  |  |  |  |  |  |  |  |
| Baldeggersee | 8.261 | 47.199 | 463 | 32.13 | 15.82 | 299.33 | NA | NA | 1.5 | 0.5 | 5 | 178 | 66 |
| Bielersee | 7.174 | 47.086 | 429 | 3.12 | 20.09 | 270.5 | NA | NA | 4.5 | 1.4 | 37 | 1120 | 74 |
| Brienzersee | 7.966 | 46.728 | 564 | 0.96 | 17.83 | 151.83 | NA | NA | 3 | 4.4 | 30 | 5170 | 261 |
| Burgaschisee | 7.668 | 47.169 | 465 | 6.06 | 19.38 | 307.17 | NA | NA | 4 | 1.3 | 0 | 2.5 | 31 |
| Caumasee | 9.296 | 46.82 | 997 | 1.64 | 16.9 | 295 | NA | NA | 7 | 1 | 0 | 0.65 | 30 |
| Greifensee | 8.677 | 47.353 | 435 | 9.98 | 21.9 | 415.33 | NA | NA | 2.5 | 1.9 | 8 | 148 | 32 |
| Hallwiesersee | 8.211 | 47.297 | 449 | 1.74 | 23.06 | 306 | NA | NA | 6 | 4.2 | 10 | 280 | 47 |
| Inkwielersee | 7.662 | 47.198 | 461 | 37.46 | 17.75 | 419.4 | NA | NA | 1.5 | 2.1 | 0 | 215 | 6 |
| Lag Grond | 9.257 | 46.808 | 1016 | 11.15 | 17.41 | 259.3 | NA | NA | 1.5 | 1 | 0 | 0.03 | 5 |
| *Melchsee | 8.273 | 46.773 | 1891 | 2.82 | 17.58 | 164.1 | NA | NA | 4 | 4.4 | 0 | 4 | 18 |
| Murtensee | 7.085 | 46.932 | 429 | 10.5 | 20.73 | 357.67 | NA | NA | 2 | 1.7 | 22 | 550 | 45 |
| Neuenburgersee | 6.859 | 46.906 | 429 | 2.26 | 21.1 | 265 | NA | NA | 7 | 1.2 | 215 | 13 900 | 153 |
| Rotsee | 8.185 | 47.041 | 419 | 8.37 | 18.71 | 190.83 | NA | NA | 3 | 2.3 | 0 | 3.8 | 16 |
| Rumensee | 8.353 | 47.194 | 545 | 23.23 | 18.48 | 443.4 | NA | NA | 0.6 | 1 | 0 | 0.01 | 2 |
| Sempachersee | 8.152 | 47.144 | 505 | 4.42 | 15.31 | 248 | NA | NA | 4.5 | 1 | 14 | 660 | 87 |
| Soppensee | 8.081 | 47.09 | 596 | 6.15 | 21 | 312.5 | NA | NA | 3.5 | 1.2 | 0 | 2.9 | 28 |
| Turlersee | 7.673 | 46.713 | 558 | 0.71 | 18.27 | 227.5 | NA | NA | 6.5 | 3.2 | 48 | 6 | 22 |
| Vierwaldstattersee | 8.503 | 47.27 | 643 | 12.14 | 19.85 | 366.5 | NA | NA | 3 | 1 | 0 | 3300 | 136 |
| Zugersee | 8.577 | 47.293 | 406 | 2.62 | 14.75 | 213.33 | NA | NA | 6.2 | 1 | 68 | 11 900 | 214 |
| Zürichsee | 8.483 | 47.161 | 413 | 4.46 | 22.42 | 244.5 | NA | NA | 3 | 1 | 114 | 3174 | 198 |

## Table S2. Pearsons’s correlation between the measured environmental parameters (transformations used in Methods; abbreviations in Table 1). The upper triangle of the table gives the *r* values (bold: significant, *p* < 0.05), and the lower part shows the number of non-missing observations used for the pairwise correlations. Four outliers of the 54 lakes were excluded prior to the analysis.

|  | Alt | lake_area | lake_vol | *Z*_max_ | PO_4_ | *Z*_S_ | Chl-*a* | Cond | TP | TN |
| --- | --- | --- | --- | --- | --- | --- | --- | --- | --- | --- |
| Alt |  | 0.05 | −0.10 | −0.24 | −0.11 | **0.36** | −0.25 | **−0.43** | −0.30 | −0.34 |
| lake_area | 48 |  | **0.52** | 0.09 | 0.24 | **0.37** | **−0.36** | −0.21 | 0.01 | **0.48** |
| lake_vol | 43 | 43 |  | **0.54** | 0.33 | **0.43** | **−0.52** | **−0.45** | −0.21 | −0.02 |
| Z_max_ | 47 | 46 | 43 |  | 0.09 | 0.28 | **−0.41** | −0.31 | −0.37 | −0.27 |
| PO_4_ | 50 | 48 | 43 | 47 |  | −0.01 | −0.06 | 0.00 | **0.43** | 0.21 |
| *Z*_S_ | 50 | 48 | 43 | 47 | 50 |  | **−0.72** | **−0.42** | **−0.55** | 0.25 |
| Chl-*a* | 50 | 48 | 43 | 47 | 50 | 50 |  | **0.60** | **0.79** | **−0.18** |
| Cond | 49 | 47 | 42 | 46 | 49 | 49 | 49 |  | **0.47** | 0.38 |
| TP | 31 | 29 | 24 | 28 | 31 | 31 | 31 | 30 |  | 0.05 |
| TN | 31 | 29 | 24 | 28 | 31 | 31 | 31 | 30 | 31 |  |

## Table S3. Environmental parameters of the 31 Austrian and German lakes (3 outliers of the 34 sampled lakes were excluded prior to the analysis; see Supplemental Table S4 for whole list of lakes).

|  | Abbrev. | *n* | Min | Max | Mean | SD |
| --- | --- | --- | --- | --- | --- | --- |
| Altitude (m) | Alt | 31 | 481 | 1447 | 710.3 | 220.5 |
| Chlorophyll *a* (μg/L) | Chl-*a* | 31 | 0.79 | 13.7 | 3.6 | 3.0 |
| Temperature in the upper 3 m (°C) | T | 22 | 10 | 19.8 | 15.6 | 2.9 |
| Conductivity (μS/cm) | Cond | 30 | 105 | 329 | 216.9 | 57.0 |
| Total phosphorus concentration (μg/L) | TP | 31 | 1.04 | 12.2 | 5.1 | 2.9 |
| Total nitrogen concentration (μg/L) | TN | 31 | 204 | 2741 | 696.4 | 419.9 |
| Secchi disk transparency (m) | *Z*_S_ | 31 | 1.7 | 16.3 | 6.5 | 3.9 |
| PO_4_ concentration (μS/cm) | PO_4_ | 31 | 1.31 | 4.5 | 2.5 | 0.9 |
| Lake area (ha) | lake_area | 29 | 11 | 50 | 31.8 | 12.4 |
| Lake volume (1000 m^3^) | lake_vol | 24 | 21 | 667 070 | 109 874 | 195 413 |
| Max. depth (m) | *Z*_max_ | 28 | 5 | 125 | 45.7 | 30.5 |

## Table S4. List of lakes and the presence of zooplankton species. Asterisks denote 4 lakes that were excluded from analyses based on environmental predictors due very high/low values (see Methods).

|  | **Copepoda** | | | | | | | | | | | | | | | | | **Cladocera** | | | | | | | | | | | | | | | | |
| --- | --- | --- | --- | --- | --- | --- | --- | --- | --- | --- | --- | --- | --- | --- | --- | --- | --- | --- | --- | --- | --- | --- | --- | --- | --- | --- | --- | --- | --- | --- | --- | --- | --- | --- |
|  | Calanoida | | | | Cyclopoida | | | | | | | | | | | | |  | | | | | | | | | | | | | | | | |
|  | *Acanthodiaptomus denticornis* | *Eudiaptomus gracilis* | *Eudiaptomus graciloides* | *Mixodiaptomus laciniatus* | *Acanthocyclops americanus* | *Acanthocyclops robustus* | *Acanthocyclops vernalis* | *Cyclops abyssorum* | *Cyclops bohater* | *Cyclops strenuus* | *Cyclops vicinus* | *Diacyclops bicuspidatus* | *Eucyclops serrulatus* | *Macrocyclops albidus* | *Mesocyclops leuckarti* | *Thermocyclops crassus* | *Thermocyclops oithonoides* | *Acroperus harpae* | *Alona affinis* | *Bosmina coregoni* | *Bosmina longirostris* | *Bosmina longispina* | *Bythotrephes longimanus* | *Ceriodaphnia pulchella* | *Ceriodaphnia quadrangula* | *Chydorus sphaericus* | *Daphnia cucullata* | *Daphnia longispina* | *Diaphanosoma brachyurum* | *Leptodora kindtii* | *Streblocerus serricaudatus* | *Polyphemus pediculus* | *Scapholeberis mucronata* | *Simocephalus vetulus* |
| **Austria** |  |  |  |  |  |  |  |  |  |  |  |  |  |  |  |  |  |  |  |  |  |  |  |  |  |  |  |  |  |  |  |  |  |  |
| Almsee | 0 | 0 | 0 | 0 | 0 | 0 | 0 | 0 | 0 | 0 | 0 | 0 | 0 | 0 | 0 | 0 | 0 | 0 | 0 | 0 | 0 | 1 | 0 | 0 | 0 | 1 | 0 | 1 | 0 | 0 | 0 | 1 | 1 | 0 |
| Ausseer See | 0 | 1 | 0 | 0 | 0 | 0 | 0 | 1 | 0 | 0 | 0 | 0 | 0 | 0 | 0 | 0 | 0 | 0 | 0 | 0 | 0 | 1 | 1 | 0 | 0 | 0 | 0 | 1 | 0 | 0 | 0 | 0 | 0 | 0 |
| Erlaufsee | 0 | 1 | 0 | 0 | 0 | 0 | 0 | 0 | 0 | 0 | 1 | 0 | 0 | 0 | 0 | 0 | 0 | 0 | 0 | 0 | 0 | 1 | 1 | 0 | 0 | 0 | 0 | 1 | 0 | 0 | 0 | 1 | 0 | 0 |
| Gleinkersee | 0 | 1 | 0 | 0 | 0 | 0 | 0 | 1 | 0 | 0 | 0 | 0 | 0 | 0 | 0 | 0 | 0 | 0 | 0 | 0 | 0 | 1 | 0 | 0 | 1 | 0 | 0 | 1 | 1 | 0 | 0 | 0 | 0 | 0 |
| Grundlsee | 0 | 1 | 0 | 0 | 0 | 0 | 0 | 1 | 0 | 0 | 0 | 0 | 0 | 0 | 0 | 0 | 0 | 0 | 0 | 0 | 0 | 1 | 1 | 0 | 0 | 0 | 0 | 1 | 0 | 1 | 0 | 0 | 1 | 0 |
| Hallstättersee | 0 | 1 | 0 | 0 | 0 | 0 | 0 | 1 | 0 | 0 | 0 | 0 | 0 | 0 | 0 | 0 | 0 | 0 | 0 | 0 | 0 | 1 | 1 | 0 | 0 | 0 | 0 | 1 | 0 | 1 | 0 | 0 | 0 | 0 |
| Hechtsee | 1 | 1 | 0 | 0 | 0 | 0 | 0 | 0 | 1 | 0 | 1 | 0 | 0 | 0 | 0 | 0 | 1 | 0 | 0 | 0 | 0 | 1 | 0 | 0 | 1 | 0 | 0 | 1 | 1 | 0 | 0 | 0 | 0 | 0 |
| Hinterer Gosausee | 0 | 1 | 0 | 0 | 0 | 0 | 0 | 1 | 0 | 0 | 0 | 0 | 0 | 0 | 0 | 0 | 0 | 0 | 0 | 0 | 0 | 1 | 0 | 0 | 0 | 0 | 0 | 1 | 0 | 0 | 0 | 0 | 0 | 0 |
| Hintersteinersee | 1 | 0 | 0 | 0 | 0 | 0 | 0 | 0 | 0 | 0 | 0 | 0 | 0 | 0 | 0 | 0 | 0 | 0 | 0 | 0 | 0 | 1 | 0 | 0 | 0 | 0 | 0 | 1 | 0 | 0 | 0 | 1 | 0 | 0 |
| Hubertussee | 0 | 1 | 0 | 0 | 0 | 0 | 0 | 0 | 0 | 0 | 1 | 1 | 1 | 1 | 0 | 0 | 0 | 0 | 0 | 0 | 1 | 0 | 0 | 0 | 1 | 0 | 0 | 1 | 0 | 0 | 0 | 1 | 0 | 1 |
| Irrsee | 0 | 1 | 0 | 0 | 0 | 0 | 0 | 0 | 1 | 0 | 0 | 0 | 0 | 0 | 1 | 0 | 0 | 0 | 0 | 0 | 0 | 1 | 1 | 0 | 0 | 0 | 0 | 1 | 0 | 1 | 0 | 0 | 0 | 0 |
| Krotensee | 0 | 1 | 0 | 0 | 0 | 0 | 0 | 0 | 1 | 0 | 0 | 0 | 0 | 0 | 1 | 0 | 0 | 0 | 0 | 0 | 0 | 1 | 0 | 0 | 1 | 0 | 0 | 1 | 0 | 0 | 0 | 0 | 0 | 0 |
| Lunzer Obersee | 1 | 0 | 0 | 0 | 0 | 0 | 1 | 0 | 0 | 0 | 0 | 0 | 0 | 0 | 0 | 0 | 0 | 1 | 0 | 0 | 0 | 1 | 0 | 0 | 1 | 1 | 0 | 1 | 0 | 0 | 0 | 1 | 0 | 0 |
| Lunzer Untersee | 0 | 1 | 0 | 0 | 0 | 0 | 0 | 1 | 0 | 0 | 0 | 0 | 0 | 0 | 0 | 0 | 0 | 0 | 0 | 0 | 0 | 1 | 0 | 0 | 0 | 0 | 0 | 1 | 0 | 0 | 0 | 0 | 0 | 0 |
| Mondsee | 0 | 1 | 0 | 0 | 0 | 0 | 0 | 1 | 1 | 0 | 0 | 0 | 0 | 0 | 1 | 0 | 0 | 0 | 0 | 0 | 0 | 1 | 1 | 0 | 0 | 0 | 0 | 1 | 1 | 1 | 0 | 0 | 0 | 0 |
| Offensee | 0 | 1 | 0 | 0 | 0 | 0 | 0 | 1 | 0 | 0 | 0 | 0 | 0 | 0 | 0 | 0 | 0 | 0 | 1 | 0 | 0 | 1 | 0 | 0 | 0 | 0 | 0 | 1 | 1 | 0 | 0 | 1 | 0 | 0 |
| Reintalersee | 0 | 1 | 0 | 0 | 0 | 0 | 0 | 0 | 0 | 1 | 0 | 0 | 0 | 0 | 1 | 0 | 0 | 0 | 0 | 0 | 0 | 1 | 0 | 0 | 1 | 0 | 1 | 1 | 1 | 0 | 0 | 0 | 0 | 0 |
| *Riesachsee | 0 | 1 | 0 | 0 | 0 | 0 | 0 | 0 | 0 | 0 | 0 | 0 | 0 | 0 | 0 | 0 | 0 | 0 | 0 | 0 | 1 | 0 | 0 | 0 | 0 | 0 | 0 | 0 | 0 | 0 | 0 | 0 | 0 | 0 |
| *Schwarzensee Sölk | 0 | 0 | 0 | 0 | 0 | 0 | 0 | 1 | 0 | 0 | 0 | 0 | 0 | 0 | 0 | 0 | 0 | 0 | 0 | 0 | 1 | 0 | 0 | 0 | 0 | 0 | 0 | 0 | 0 | 0 | 0 | 0 | 0 | 0 |
| Schwarzensee Strobl | 0 | 1 | 0 | 0 | 0 | 0 | 0 | 0 | 1 | 0 | 0 | 0 | 0 | 0 | 1 | 0 | 0 | 0 | 0 | 0 | 0 | 1 | 0 | 0 | 0 | 0 | 0 | 1 | 1 | 1 | 0 | 0 | 0 | 0 |
| Steierersee | 0 | 1 | 0 | 0 | 0 | 0 | 0 | 1 | 0 | 0 | 0 | 0 | 0 | 0 | 0 | 0 | 0 | 0 | 0 | 0 | 0 | 1 | 0 | 0 | 1 | 0 | 0 | 1 | 0 | 0 | 0 | 0 | 0 | 0 |
| Toplitzsee | 0 | 1 | 0 | 0 | 0 | 0 | 0 | 1 | 0 | 0 | 0 | 0 | 0 | 0 | 0 | 0 | 0 | 0 | 0 | 0 | 0 | 1 | 1 | 0 | 0 | 0 | 0 | 1 | 1 | 0 | 0 | 0 | 0 | 0 |
| Vorderer Gosausee | 0 | 1 | 0 | 0 | 0 | 0 | 0 | 1 | 0 | 0 | 0 | 0 | 0 | 0 | 0 | 0 | 0 | 0 | 0 | 0 | 0 | 1 | 0 | 0 | 0 | 0 | 0 | 1 | 0 | 0 | 0 | 0 | 1 | 0 |
| Walchsee | 0 | 1 | 0 | 0 | 0 | 0 | 0 | 0 | 0 | 0 | 0 | 0 | 0 | 0 | 1 | 0 | 0 | 0 | 0 | 0 | 0 | 1 | 0 | 0 | 1 | 1 | 0 | 1 | 1 | 0 | 0 | 0 | 0 | 0 |
| Wallersee | 0 | 1 | 0 | 0 | 1 | 0 | 0 | 1 | 0 | 0 | 0 | 0 | 0 | 0 | 1 | 1 | 0 | 0 | 0 | 0 | 0 | 0 | 0 | 0 | 0 | 0 | 0 | 1 | 1 | 1 | 0 | 0 | 0 | 0 |
| Wolfgangsee | 0 | 1 | 0 | 0 | 0 | 0 | 0 | 1 | 0 | 0 | 0 | 0 | 0 | 0 | 1 | 0 | 0 | 0 | 0 | 0 | 0 | 1 | 1 | 0 | 0 | 0 | 0 | 1 | 1 | 1 | 0 | 0 | 0 | 0 |
|  |  |  |  |  |  |  |  |  |  |  |  |  |  |  |  |  |  |  |  |  |  |  |  |  |  |  |  |  |  |  |  |  |  |  |
| **Germany** |  |  |  |  |  |  |  |  |  |  |  |  |  |  |  |  |  |  |  |  |  |  |  |  |  |  |  |  |  |  |  |  |  |  |
| Brunnsee | 0 | 1 | 1 | 0 | 0 | 0 | 0 | 0 | 1 | 0 | 0 | 0 | 0 | 0 | 1 | 0 | 1 | 0 | 0 | 0 | 0 | 0 | 0 | 0 | 1 | 0 | 0 | 1 | 0 | 1 | 0 | 0 | 0 | 0 |
| Kirchsee | 0 | 1 | 0 | 0 | 0 | 0 | 0 | 0 | 0 | 0 | 0 | 0 | 0 | 0 | 1 | 0 | 0 | 0 | 0 | 0 | 0 | 1 | 0 | 0 | 1 | 0 | 0 | 1 | 1 | 0 | 0 | 0 | 0 | 0 |
| Klostersee | 0 | 1 | 1 | 0 | 0 | 0 | 0 | 0 | 1 | 0 | 0 | 0 | 0 | 0 | 1 | 1 | 1 | 0 | 0 | 0 | 0 | 1 | 0 | 0 | 1 | 1 | 0 | 1 | 1 | 1 | 0 | 0 | 0 | 0 |
| Langbürgner See | 0 | 1 | 1 | 0 | 0 | 0 | 0 | 1 | 0 | 0 | 0 | 0 | 0 | 0 | 1 | 0 | 0 | 0 | 0 | 0 | 0 | 1 | 0 | 0 | 1 | 0 | 0 | 1 | 1 | 0 | 0 | 0 | 0 | 0 |
| Schliersee | 0 | 0 | 0 | 0 | 0 | 0 | 0 | 1 | 0 | 0 | 0 | 0 | 0 | 0 | 0 | 0 | 0 | 0 | 0 | 0 | 0 | 0 | 0 | 0 | 0 | 0 | 0 | 0 | 0 | 0 | 0 | 0 | 0 | 0 |
| *Spitzingsee | 0 | 1 | 0 | 0 | 0 | 0 | 0 | 1 | 0 | 0 | 0 | 0 | 0 | 0 | 0 | 0 | 1 | 0 | 0 | 0 | 0 | 1 | 0 | 0 | 0 | 0 | 0 | 1 | 0 | 1 | 0 | 0 | 0 | 0 |
| Tegernsee | 0 | 1 | 0 | 0 | 0 | 0 | 0 | 1 | 0 | 0 | 0 | 0 | 0 | 0 | 1 | 0 | 0 | 0 | 0 | 0 | 0 | 1 | 1 | 0 | 0 | 0 | 0 | 1 | 1 | 1 | 0 | 0 | 0 | 0 |
| Weitsee | 0 | 0 | 0 | 0 | 0 | 0 | 0 | 0 | 0 | 0 | 0 | 0 | 0 | 0 | 1 | 0 | 0 | 0 | 1 | 0 | 0 | 1 | 0 | 0 | 1 | 0 | 0 | 1 | 0 | 0 | 1 | 0 | 0 | 0 |
|  |  |  |  |  |  |  |  |  |  |  |  |  |  |  |  |  |  |  |  |  |  |  |  |  |  |  |  |  |  |  |  |  |  |  |
| **Switzerland** |  |  |  |  |  |  |  |  |  |  |  |  |  |  |  |  |  |  |  |  |  |  |  |  |  |  |  |  |  |  |  |  |  |  |
| Baldeggersee | 0 | 1 | 0 | 0 | 0 | 0 | 0 | 1 | 0 | 0 | 0 | 0 | 0 | 0 | 1 | 1 | 0 | 0 | 0 | 0 | 0 | 0 | 0 | 0 | 0 | 0 | 0 | 1 | 1 | 1 | 0 | 0 | 0 | 0 |
| Bielersee | 0 | 1 | 0 | 0 | 0 | 0 | 0 | 1 | 0 | 0 | 1 | 0 | 0 | 0 | 1 | 1 | 0 | 0 | 0 | 1 | 0 | 0 | 0 | 0 | 0 | 0 | 1 | 1 | 1 | 0 | 0 | 0 | 0 | 0 |
| Brienzersee | 0 | 1 | 0 | 0 | 0 | 0 | 0 | 1 | 0 | 0 | 0 | 0 | 0 | 0 | 0 | 0 | 0 | 0 | 0 | 0 | 0 | 1 | 0 | 0 | 0 | 0 | 0 | 1 | 1 | 1 | 0 | 0 | 1 | 0 |
| Burgaschisee | 0 | 1 | 0 | 0 | 0 | 0 | 0 | 0 | 0 | 0 | 1 | 0 | 0 | 0 | 1 | 1 | 0 | 0 | 0 | 0 | 0 | 0 | 0 | 1 | 0 | 0 | 0 | 1 | 1 | 0 | 0 | 0 | 0 | 0 |
| Caumasee | 0 | 0 | 0 | 0 | 0 | 0 | 0 | 1 | 0 | 0 | 0 | 0 | 0 | 0 | 0 | 0 | 0 | 0 | 0 | 0 | 0 | 1 | 0 | 0 | 0 | 0 | 0 | 1 | 0 | 0 | 0 | 0 | 0 | 0 |
| Greifensee | 0 | 1 | 0 | 0 | 0 | 0 | 0 | 1 | 0 | 0 | 0 | 0 | 0 | 0 | 1 | 0 | 0 | 0 | 0 | 0 | 0 | 0 | 1 | 0 | 0 | 0 | 0 | 1 | 0 | 1 | 0 | 0 | 0 | 0 |
| Hallwiesersee | 0 | 1 | 0 | 0 | 0 | 0 | 0 | 1 | 0 | 0 | 0 | 0 | 0 | 0 | 1 | 1 | 0 | 0 | 0 | 1 | 0 | 0 | 0 | 0 | 0 | 0 | 0 | 1 | 1 | 0 | 0 | 0 | 0 | 0 |
| Inkwielersee | 0 | 0 | 0 | 0 | 0 | 0 | 0 | 0 | 0 | 0 | 0 | 0 | 0 | 0 | 1 | 0 | 0 | 0 | 0 | 0 | 1 | 0 | 0 | 0 | 0 | 0 | 0 | 1 | 0 | 0 | 0 | 0 | 0 | 0 |
| Lag Grond | 0 | 1 | 0 | 0 | 0 | 1 | 0 | 1 | 0 | 0 | 0 | 0 | 0 | 0 | 1 | 0 | 0 | 0 | 0 | 0 | 1 | 0 | 0 | 0 | 0 | 0 | 0 | 1 | 0 | 0 | 0 | 0 | 0 | 0 |
| *Melchsee | 0 | 1 | 0 | 0 | 0 | 0 | 1 | 0 | 0 | 0 | 0 | 0 | 1 | 0 | 0 | 0 | 0 | 1 | 0 | 0 | 1 | 0 | 0 | 0 | 0 | 1 | 0 | 1 | 0 | 0 | 0 | 0 | 0 | 0 |
| Murtensee | 0 | 1 | 0 | 0 | 0 | 0 | 0 | 1 | 0 | 0 | 0 | 0 | 0 | 0 | 1 | 1 | 0 | 0 | 0 | 0 | 1 | 0 | 0 | 0 | 0 | 0 | 0 | 1 | 1 | 0 | 0 | 0 | 0 | 0 |
| Neuenburgersee | 0 | 1 | 0 | 0 | 0 | 0 | 0 | 1 | 0 | 0 | 0 | 0 | 0 | 0 | 1 | 0 | 0 | 0 | 0 | 1 | 0 | 0 | 0 | 0 | 0 | 0 | 1 | 0 | 1 | 1 | 0 | 0 | 0 | 0 |
| Rotsee | 0 | 1 | 0 | 0 | 0 | 0 | 0 | 1 | 0 | 0 | 0 | 0 | 0 | 0 | 1 | 0 | 0 | 0 | 0 | 0 | 0 | 0 | 0 | 0 | 0 | 0 | 0 | 1 | 1 | 0 | 0 | 0 | 0 | 0 |
| Rumensee | 0 | 0 | 0 | 0 | 0 | 0 | 0 | 1 | 0 | 0 | 0 | 0 | 0 | 0 | 1 | 0 | 0 | 0 | 0 | 0 | 1 | 0 | 0 | 1 | 0 | 0 | 0 | 0 | 0 | 0 | 0 | 0 | 0 | 0 |
| Sempachersee | 0 | 1 | 0 | 0 | 0 | 0 | 0 | 1 | 0 | 0 | 0 | 0 | 0 | 0 | 1 | 0 | 0 | 0 | 0 | 0 | 0 | 1 | 0 | 0 | 0 | 0 | 1 | 0 | 1 | 1 | 0 | 0 | 0 | 0 |
| Soppensee | 0 | 1 | 0 | 0 | 0 | 0 | 0 | 0 | 0 | 0 | 0 | 0 | 0 | 0 | 1 | 0 | 0 | 0 | 0 | 0 | 0 | 0 | 0 | 0 | 0 | 0 | 0 | 1 | 0 | 0 | 0 | 0 | 0 | 0 |
| Turlersee | 0 | 1 | 0 | 0 | 0 | 0 | 0 | 1 | 0 | 0 | 0 | 0 | 0 | 0 | 1 | 0 | 0 | 0 | 0 | 0 | 0 | 1 | 0 | 0 | 0 | 0 | 0 | 1 | 0 | 1 | 0 | 0 | 0 | 0 |
| Vierwaldstattersee | 0 | 1 | 0 | 0 | 0 | 0 | 0 | 1 | 0 | 0 | 0 | 0 | 0 | 0 | 1 | 0 | 0 | 0 | 0 | 0 | 0 | 0 | 1 | 0 | 0 | 0 | 0 | 1 | 1 | 1 | 0 | 0 | 0 | 0 |
| Zugersee | 0 | 1 | 0 | 1 | 0 | 0 | 0 | 1 | 0 | 0 | 0 | 0 | 0 | 0 | 1 | 0 | 0 | 0 | 0 | 0 | 0 | 0 | 1 | 0 | 0 | 0 | 0 | 1 | 0 | 1 | 0 | 0 | 0 | 0 |
| Zürichsee | 0 | 1 | 0 | 0 | 0 | 0 | 0 | 1 | 0 | 0 | 0 | 0 | 0 | 0 | 1 | 0 | 0 | 0 | 0 | 0 | 0 | 1 | 1 | 0 | 0 | 0 | 0 | 1 | 0 | 1 | 0 | 0 | 0 | 0 |
| **No. of occurrences** | 3 | 45 | 3 | 1 | 1 | 1 | 2 | 34 | 7 | 1 | 5 | 1 | 2 | 1 | 31 | 7 | 4 | 2 | 2 | 3 | 8 | 33 | 13 | 2 | 13 | 5 | 4 | 48 | 24 | 20 | 1 | 6 | 4 | 1 |

## Table S5. Pearsons’s correlation between the measured environmental parameters of 31 Austrian and German lakes (transformations used, see Methods; abbreviations listed in Table 1). The upper triangle of the table gives the *r* values (bold: significant, *p* < 0.05), whereas the lower part shows the number of non-missing observations used for the pairwise correlations. Three outliers of the 34 sampled lakes were excluded prior to the analysis; whole list of lakes is in Supplemental Table S4).

|  | Alt | lake_area | lake_vol | *Z*_max_ | PO_4_ | *Z*_S_ | Chl-*a* | Cond | TP | TN |
| --- | --- | --- | --- | --- | --- | --- | --- | --- | --- | --- |
| Alt |  | −0.01 | −**0.43** | 0.11 | −0.3 | **0.36** | −0.11 | **−0.46** | −0.3 | −0.34 |
| lake_area | 29 |  | −0.13 | **−0.43** | 0.11 | 0.22 | −0.25 | 0.2 | 0.01 | **0.48** |
| lake_vol | 24 | 24 |  | **0.76** | −0.01 | −0.16 | −0.34 | 0.19 | −0.21 | −0.02 |
| *Z*_max_ | 28 | 27 | 23 |  | −0.31 | 0.2 | −0.3 | −0.19 | −0.37 | −0.27 |
| PO_4_ | 31 | 29 | 24 | 28 |  | **−0.41** | **0.59** | **0.61** | **0.43** | 0.21 |
| *Z*_S_ | 31 | 29 | 24 | 28 | 31 |  | **−0.55** | −0.18 | **−0.55** | 0.25 |
| Chl-*a* | 31 | 29 | 24 | 28 | 31 | 31 |  | **0.4** | **0.79** | −0.18 |
| Cond | 30 | 28 | 23 | 27 | 30 | 30 | 30 |  | **0.47** | **0.38** |
| TP | 31 | 29 | 24 | 28 | 31 | 31 | 31 | 30 |  | 0.05 |
| TN | 31 | 29 | 24 | 28 | 31 | 31 | 31 | 30 | 31 |  |

## Table S6. Occurrence of *Bythotrephes longimanus* since the 1990s in the Alps (▲ in Fig. 1). We only included lakes not sampled in 2011–2012.

| **Country** | **Lake** | **Latitude** | **Longitude** | **Source** | **Year** |
| --- | --- | --- | --- | --- | --- |
| Austria | Altausseer See | 47.641534 | 13.785321 | Gaviria-Melo et al. (2005) | 2000 |
| Austria | Attersee | 47.877322 | 13.545667 | Gaviria-Melo et al. (2005) | 1999 |
| Austria | Fuschlsee | 47.803776 | 13.275585 | Gaviria-Melo et al. (2005) | 1998 |
| Austria | Traunsee | 47.872799 | 13.793491 | Gaviria-Melo et al. (2005) | 1999 |
| Germany | Lake Constance | 47.648582 | 9.390658 | Palmer et al. (2001) | 1998 |
| Germany | Müggelsee | 52.437268 | 13.650407 | Therriault et al. (2002) | 1991 |
| Italy | Lago d'Iseo | 45.727883 | 10.065877 | Therriault et al. (2002) | NA |
| Italy | Lago di Como | 46.030373 | 9.253289 | Therriault et al. (2002) | NA |
| Italy | Lago di Garda | 45.63594 | 10.630646 | Therriault et al. (2002) | NA |
| Italy | Lago di Lugano | 45.993045 | 8.968595 | Therriault et al. (2002) | NA |
| Italy | Lago di Mergozzo | 45.955702 | 8.464899 | Therriault et al. (2002) | NA |
| Italy | Lago Maggiore | 46.057624 | 8.627602 | Manca (2011) | 1995 |
| Switzerland | Lake Lucerne | 47.020272 | 8.434726 | Enz et al. (2001) | 1994 |
| Switzerland | Walensee | 47.123596 | 9.201788 | Therriault et al. (2002) | NA |

## Figure S1. Standardised PCA plot of environmental features of the lakes (for abbreviations, see Table 1). Ordination is based on 41 observations (4 outliers and 9 samples with missing data were excluded, see Methods).

# References

Enz CA, Heller C, Müller R, Bürgi H-R. 2001. Investigations on fecundity of *Bythotrephes longimanus* in Lake Lucerne (Switzerland) and on niche segregation of *Leptodora kindti* and *Bythotrephes longimanus* in Swiss lakes. Hydrobiologia. 464:143–151.

Gaviria-Melo S, Forró L, Jersabek CD, Schabetsberger R. 2005. Checklist and distribution of cladocerans and leptodorans (Crustacea: Branchiopoda) from Austria. Ann Naturhist Mus Wien. 106:145–216.

Manca M. 2011. Invasions and re-emergences: an analysis of the success of *Bythotrephes* in Lago Maggiore (Italy). J Limnol. 70:76–82.

Palmer A, Stich H-B, Maier G. 2001. Distribution patterns and predation risk of the coexisting cladocerans *Bythotrephes longimanus* and *Leptodora kindtii* in a large lake–Lake Constance. Hydrobiologia. 442:301–307.

Therriault TW, Grigorovich IA, Cristescu ME, Ketelaars HAM, Viljanen M, Heath DD, Macisaac HJ. 2002. Taxonomic resolution of the genus *Bythotrephes* Leydig using molecular markers and re-evaluation of its global distribution. Divers Distrib. 8:67–84.
